# Analysis of economic burden and its associated factors of twenty-three rare diseases in Shanghai

**DOI:** 10.1186/s13023-019-1168-4

**Published:** 2019-10-22

**Authors:** Xiaoshu Cai, Hui Yang, Georgi Z. Genchev, Hui Lu, Guangjun Yu

**Affiliations:** 1 Center for Biomedical Informatics, Shanghai Children’s Hospital, Shanghai, China; 2 Walkiesoft Ltd, Shanghai, China; 3 SJTU-Yale Joint Center for Biostatistics, Shanghai Jiaotong University, Shanghai, China; 4 Bulgarian Institute of Genomics and Precision Medicine, Sofia, Bulgaria; 5 Department of Bioengineering, University of Illinois at Chicago, Chicago, USA; 6 Department of Bioinformatics and Biostatistics, Shanghai Jiaotong University, Shanghai, China

**Keywords:** Rare disease, Economic burden, Shanghai, Cost of illness

## Abstract

**Background:**

It is estimated that at present there are over 10 million rare disease patients in China. Recently an increased focus from policy perspective has been placed on rare diseases management. Improved disease definitions and the releases of local and national rare disease lists are some of the steps taken already. Despite these developments, few Chinese rare disease-related epidemiology and economic studies exist, thus hindering assessment of the true burden of rare diseases. For a rare disease with an effective treatment, this is a particularly important aspect due to the often-high cost associated.

**Objective:**

The goal of this study is to address the data scarcity on the subject of rare diseases economic impact in China. We aim to address an existing knowledge gap and to provide a timely analysis of the economic burden of 23 rare diseases in Shanghai, China.

**Methods:**

We utilized the data from the Health Information Exchange system of Shanghai and employed statistical modeling to analyze the economic burden of rare diseases with an effective treatment in Shanghai.

**Results:**

First, we described the actual direct medical expenditure and analyzed its associated factors. Second, we found age, disease type, number of complications, and payment type were significantly associated with rare disease medical direct costs. Third, a generalized linear model was employed to estimate the annual direct cost. The mean direct medical cost was estimated as ¥9588 (US$1521) for inpatients and ¥1060 (US$168) for outpatients, and was over ¥15 million (~US$2.4 million) per year overall.

**Conclusion:**

Our study is one of the first quantifying the economic burden of an extensive set of rare diseases in Shanghai and China. Our results can serve to inform healthcare-focused policy making, contribute to the increase of public awareness, and incentivize development of rare-disease strategies and treatments specific to the Chinese context.

**Electronic supplementary material:**

The online version of this article (10.1186/s13023-019-1168-4) contains supplementary material, which is available to authorized users.

## Background

A rare disease is generally a condition that affects a very small percentage of the population. The World Health Organization (WHO) defines rare diseases as one with prevalence between 0.65–1‰, however some countries use different definitions [1]. For example in the USA, a disease is defined as rare when it affects less than 200,000 people; in the European Union - when it affects less than 1 in 2000 people.

Rare diseases are mostly related to uncertain pathogenesis, less than 1% of them have effective treatments [2] and effective treatments can be very costly. Frequently, patients suffer from diagnostic delays, inadequate management, and lack of information and resources. For a rare disease with effective treatments, there is especially a strong health interest relating to their economic burden. Recent studies have reported economic burden of diseases such as hemophilia [3–5], cystic fibrosis [6, 7], phenylketonuria [8], and fragile X syndrome [9, 10] from the perspective of patients, families, and societies in Europe, United States, and Canada.

China has the largest cumulative number of patients with rare diseases, and according to recent World Health Organization estimates, there are over 10 million rare disease patients in China [11]. As of 2018, while measures are being taken to address existing challenges, China is still in the early stages of developing a comprehensive rare disease policy. Awareness of rare diseases in China has received increased attention in recent years but as of yet China has not officially established a definition of rare disease due to a lag in legislation and stakeholder consensus [12, 13]. A list-based approach has been implemented in one of the Chinese major cities - in 2016, the Shanghai Health and Family Planning Commission released *The List of Major Rare Diseases in Shanghai* [14] which was the first local list of rare diseases in China .

While rare disease research has received increased focus of attention in China, few epidemiology and economic studies exits, and research offering a comprehensive analysis of rare diseases burden is largely absent in China, thus hindering assessment of the true burden of rare diseases [15]. Data scarcity caused by gaps in the patient registration systems is one of the reasons and furthermore, existing current studies cover a limited proportion of rare diseases which have effective treatment. For example, in contrast to US and EU [16, 17], there are no China-focused studies with information on the cost of illness related to the Prader-Willi Syndrome - a well-known genetic pediatric disease. Given the potentially large number of individuals with rare diseases, and the lack of focused studies, there is a pressing need to investigate the rare diseases economic burden and socio-economic impact as a reference and input to health policy and regulatory development.

This study focuses on the Shanghai rare diseases list and provides much-needed analysis of the economic burden, focusing on direct medical cost in rare diseases in Shanghai. Our work is amongst the first that analyzes the economic burden of a publicly released set of rare diseases in one of the China’s first tier cities. Through discovering the state of rare disease burden in Shanghai, our study aims to fill the gap in rare disease economic research and present useful evidence for policy making in China. Thus, our results can be utilized in the design of a comprehensive rare disease policy specific to the Chinese context.

## Methods

Herein we present a cost of illness cross sectional study which takes the perspective of the health system participants and is thus focused on direct medical costs.

### Data source

Rare disease data was collected from the Health Information Exchange (HIE) system in Shanghai. The HIE system of Shanghai was established by the Shanghai Hospital Development Center in 2010, integrating medical records from 38 tertiary hospitals and 40 community health centers in Shanghai. The HIE system contains over 210 million visit records, 16 million prescriptions, 9.9 million case notes, and 230 million laboratory results, with coverage of 61 million patients [18]. The Health Information Exchange (HIE) system is based on a standard framework and provides the data foundation for hospital business analytics, operations, and financials to name a few. Additionally it can inform quality control and patient management, and provides an extensive source of healthcare data for academic research and analysis.

Recently, the National Health and Family Planning Commission of Shanghai published a list of 56 rare diseases with effective treatments [14]. Taking the Shanghai list (see Additional file 1: Table S1) as a base, we mapped disease names from the list to a standard ICD10 code (International Statistical Classification of Disease and Related Health Problems 10th Shanghai Revision), which is used by most tertiary hospitals in Shanghai. We extracted medical information for 34 rare diseases from the Shanghai list from 01/2013 to 12/2016; the rest of diseases did not have a matching code and no health record information could be identified in the HIE system. The rare disease data obtained from the HIE system consisted of medical records containing patient demographic information, outpatient records, inpatient records, prescriptions, doctor’s advice, diagnostic records, indicators, radiology information system reports, and hospital discharge records.

### Data extraction and processing

The medical records obtained contain major diagnosis and several secondary diagnoses for every patient. Patients with either major diagnosis or secondary diagnosis were considered as our target population. From the medical records, we extracted a feature set containing patient medical record number, gender, date of birth, diagnosis and corresponding ICD10 code, number of complications, and direct medical cost. Direct medical cost consisted of 16 item categories such as registration, hospitalization, diagnostic, treatment cost and so on (see Additional file 1: Table S2). Within the set of 34 diseases that we mapped to ICD-10 codes, there were 11 diseases that did not have cost information available on the HIE system of Shanghai (see Additional file 1: Table S3). These 11 diseases were not included in the calculations and the modeling.

In addition, medical costs were combined if a patient had more than one medical record. For the disease hemophilia, which has multiple subtypes, we aggregated all subtypes: hemophilia (ICD = D66.× 02), hemophilia A (ICD = D66.× 01), hemophilia B (ICD = D67.× 01), hemophilia C (ICD = D68.101). We assigned each disease to one of the following disease categories: endocrine and metabolic disease, skin disease, blood disease, digestive disease, bone disease, cardiovascular disease, immunological disease, and kidney disease. We also considered the payment types: social security card (*shebaoka*,社保卡) and medicare card (*yibaoka*, 医保卡) both of which provide cost reimbursement via a medical insurance coverage scheme; and the two self-funded types – the hospital link card (*yilianka*, 医联卡) and the hospital self-charge card (*zifeika*, 自费卡) (see Additional file 1: Table S4). To protect the privacy of patients, personally identifiable information was obfuscated. Data were stored in a MySQL relational database and processed with the database software DbVisualizer (DbVis Software AB, Stockholm, Sweden) and by using SQL.

### Statistical analysis

Statistical analysis was carried out using IBM SPSS Statistics v23 software (IBM Corporation, USA). Normality of distribution was assessed through the One-Sample Kolmogorov-Smirnov Test for all the variables. The various medical costs were expressed as medians and interquartile ranges [IQR]. In the univariate analysis, comparisons of continuous variables were made by using Mann–Whitney U tests (for 2 groups) or Kruskal-Wallis H tests (for multiple groups); categorical variables were presented as frequency (percentage) and compared using the chi-square test as appropriate. To estimate the medical cost, multivariable generalized linear models (gamma with log link) were implemented on the cost of inpatients and outpatients, respectively. The model was employed to analyze the association between selected variables and direct medical costs. The model coefficients for each variable were predicted, and *p*-value was calculated for coefficient B. The model’s goodness of fit was compared using deviance and chi-square (per degree of freedom) to choose the best model. The selected best model was used to estimate cost per applicable features and the grand mean of annual medical costs of patients with rare diseases. 95% confidence intervals around the means of the estimations were generated. Analogously, *p*-value was also calculated for comparing estimated means with observed means. Statistical significance was set to 5%. For the currency exchange rate, we employed US $1 = Chinese yuan ¥6.30, calculated as the period average of the exchange rate published by The People’s Bank of China.

## Results

In the work, we employed a large-scale data analysis of patient data obtained from the Shanghai HIE system. First, we developed a demographical description and clinical characterization. Second, we described the actual direct cost of 23 rare diseases with effective treatment defined in the Shanghai rare disease list, and found that age, disease type, number of complications, and payment types were significantly associated with economic burden imposed. Finally, we implemented a generalized linear model to estimate the direct medical cost for inpatients, outpatients, and overall.

### Demographic and clinical characteristics

Regarding the 23 rare diseases, from January 2013 to December 2016 there were total of 16,933 patients diagnosed; of which 5185(30.6%) were inpatients and 11,748(69.4%) were outpatients. Among them, children (age ≤ 14) and seniors (age > 65) were a minor population group in this data collection, 25.2 and 10.9% respectively. Male patients accounted for 75.1%. The unbalanced gender distribution was due to the existence of records where gender information was not entered or not available. Sample size and mean cost for each disease are shown in Additional file 1: Table S3. Every disease was assigned to a disease category. Among the eight disease category types (endocrine and metabolic disease, skin disease, blood disease, digestive disease, bone disease, cardiovascular disease, immunological disease, and kidney disease) blood disease category accounted for 46.8%. The blood disease category contained rare diseases with large population, such as hemophilia and severe congenital neutropenia. Number of complications can be considered as an indication of disease severity. A total of 57% inpatients and 43% outpatients were experiencing more than one complication. Of the patients experiencing complications, 2.3% of patients are severe with over 10 complications. Patients using social security card (*shebaoka*, 社保卡) and medicare card (*yibaoka*, 医保卡), were 31.80 and 15.71%, with approximately half of the patients being able to get reimbursement from public medical insurance. Table 1 shows full demographic description of the dataset.

**Table 1:** Demographic and clinical characteristics of patients with rare diseases according to subgroups [n(%)]

	Inpatients	Outpatients	Total
Age group (years)			
≤ 14	816 (34.23%)	2632 (23.32%)	3448 (25.2%)
15~64	1287 (53.98%)	7443 (65.94%)	8730 (63.9%)
≥ 65	281 (11.79%)	1213 (10.74%)	1494 (10.9%)
Gender			
Male	5292 (75.01%)	1946 (75.40%)	7248 (75.1%)
Female	1763 (24.99%)	635 (24.60%)	2403 (24.9%)
Disease type			
Endocrine and metabolic disease	4479 (36.89%)	879 (23.96%)	5358 (33.86%)
Skin disease	128 (1.07%)	45 (1.23%)	173 (1.09%)
Blood disease	5667 (46.76%)	1715 (46.74%)	7406 (46.80%)
Digestive disease	1300 (10.71%)	315 (8.59%)	1616 (10.21%)
Bone disease	115 (0.95%)	36 (0.98%)	151 (0.95%)
Cardiovascular disease	423 (3.48%)	654 (17.83%)	1077 (6.81%)
Immunological disease	14 (0.12%)	14 (0.38%)	28 (0.18%)
Kidney disease	5 (0.04%)	5 (0.30%)	16 (0.10%)
Number of complications			
1	4947 (64.19%)	787 (27.59%)	5734 (54.24%)
2	1791 (23.24%)	480 (16.83%)	2272 (21.49%)
3	607 (7.88%)	378 (13.25%)	994 (9.40%)
4	151 (1.96%)	295 (10.34%)	446 (4.22%)
5	74 (0.96%)	234 (8.20%)	309 (2.92%)
6	32 (0.42%)	187 (6.56%)	220 (2.08%)
7	27 (0.35%)	126 (4.42%)	153 (1.45%)
8	15 (0.19%)	107 (3.75%)	122 (1.15%)
9	14 (0.19%)	64 (2.24%)	79 (0.75%)
> 10	49 (0.64%)	194 (6.80%)	243 (2.30%)
Payment type			
Social Security Card	4193 (34.79%)	522 (18.89%)	4715 (31.80%)
Medicare Card	2013 (16.70%)	313 (11.33%)	2330 (15.71%)
Hospital Link Card	5517 (45.78%)	1862 (67.39%)	7388 (49.82%)
Hospital Self-Charge Card	324 (2.69%)	37 (1.34%)	361 (2.43%)
Others	5 (0.04%)	29 (1.05%)	34 (0.23%)

**Fig. 1:**
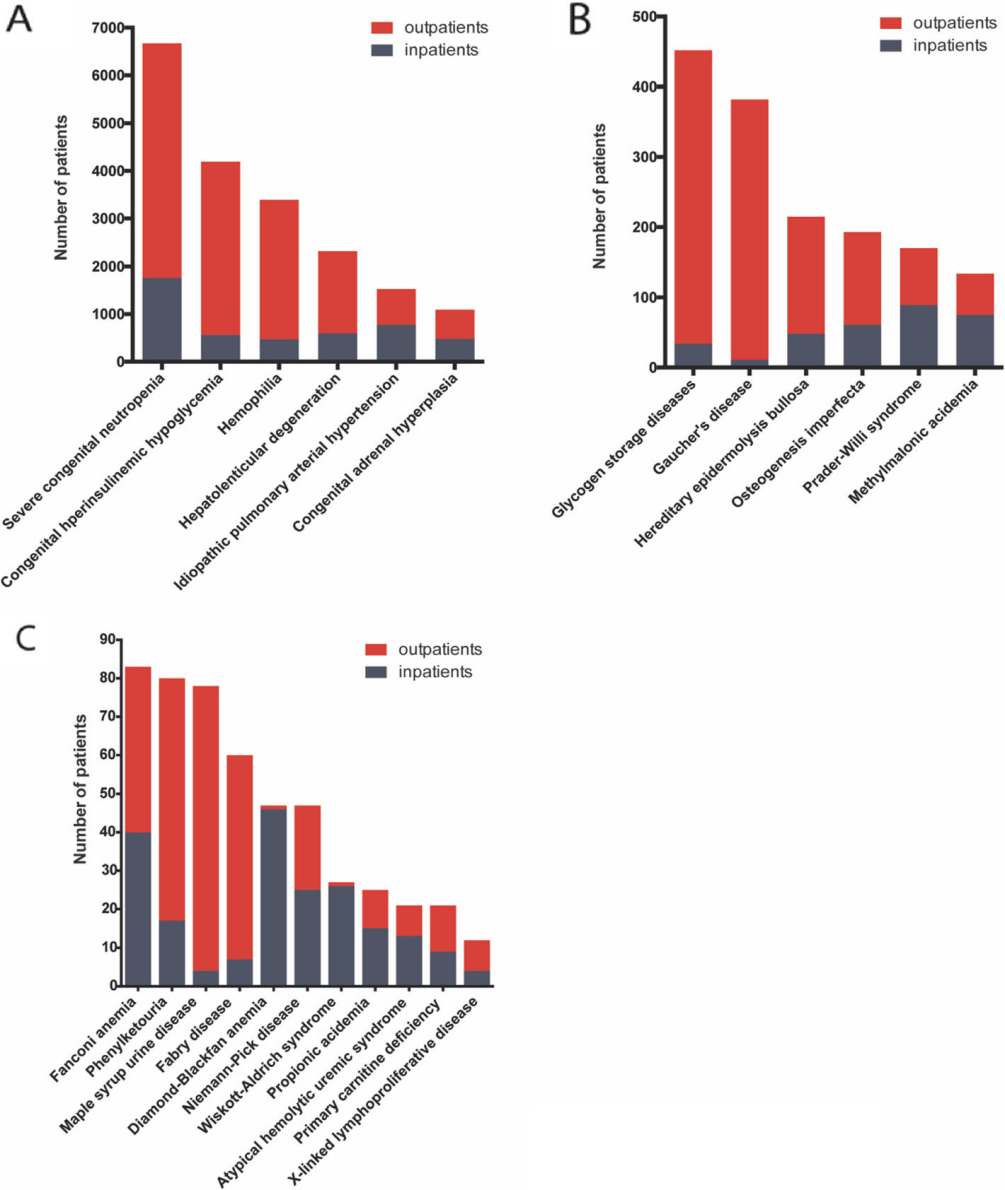
Number of inpatients and outpatients with rare diseases. Panel **a**: Number of patients - larger than 1000; Panel **b**: Number of patients - within the range of 100-1000; Panel **c**: Number of patients - less than 100

There is a notable difference in the numbers of inpatients and outpatients across the 23 rare diseases. Generally, outpatients accounted for a greater proportion in a majority of diseases (Fig. 1). In 2 isolated cases, Diamond-Blackfan anemia and Wiscott-Aldrich syndrome, inpatient number vastly outnumbered outpatients. In a few other cases, the proportions of inpatients and outpatients were nearly equal.

### Univariate medical cost analysis

The mean cost of annual medical expenditure was ¥9588.27 (US$1521.94) among inpatients and ¥1060.28 (US$168.29) among outpatients. The mean cost was far surpassing the median cost (¥2952.93 in inpatients and ¥79.27 in outpatients), indicating a right-skewed distribution of direct medical cost in rare diseases, therefore we implemented a rank sum test for the univariate analysis. The result showed that even though the population of outpatients is much larger than inpatients admitted to hospital for a rare disease treatment, the average cost per person of inpatients is approximately 10 times the outpatient cost.

We analyzed the effects of the feature set factors (age, gender, number of complication, disease category, and payment type) on inpatient and outpatient costs (Table 2). Comparing patients of different age groups, seniors (age ≥ 65) had a significant higher cost than children (age ≤ 14) and adults (14 < age < 65). Significant difference was not found among inpatients in different gender (*p* = 0.083). Significantly higher inpatient costs were observed in patients with kidney disease, who had a median cost of ¥12,529.70 (¥3792.96–¥16,518.54). Experiencing a larger number of complications was responsible for a higher medical cost (*p* < 0.001). Generally, inpatients medical cost was higher than outpatients cost (z = − 88.416, *p* < 0.001) but in case when patients experienced more than 6 complications, costs in outpatient groups were higher (*p* < 0.001). Finally, we found that patients with hospital self-charge card (*zifeika*, 自费卡) had a lower expenditure than patients with social security card (*shebaoka*, 社保卡) (*p* = 0.039), due to some extent to restrictions on medication and testing on self-charge items.

**Table 2:** Univariate analysis of the factors associated with annual direct cost (¥) of patients with rare disease [Median (P_25_-P_75_)]

	Inpatients costs	Outpatients costs	Z	P
Age group (years)				
≤ 14	2500 (1195–5778)	82 (27–296)	−37.777	< 0.001
15~64	3604 (1585–11,121)	75 (28–209)	−51.787	< 0.001
≥ 65	4104 (2300–7138)	326 (171–781)	−24.486	< 0.001
P	< 0.001	< 0.001		
Gender				
Male	3103 (1414–8156)	102 (33–324)	−55.002	< 0.001
Female	2474 (1302–5559)	77 (29–204)	−34.530	< 0.001
p	0.083	< 0.001		
Disease type				
Endocrine and metabolic disease	1757 (1055–2980)	60 (24–145)	−43.484	< 0.001
Skin disease	3619 (2410–5971)	17 (12–114)	−9.624	< 0.001
Blood disease	3337 (1405–12,208)	92 (33–303)	−53.333	< 0.001
Digestive disease	318 (2025–5756)	141 (59–352)	−25.282	< 0.001
Bone disease	4976 (1755–8913)	145 (63–277)	− 8.597	< 0.001
Cardiovascular disease	4686 (2652–7390)	78 (37–225)	− 24.458	< 0.001
Immunological disease	9228 (173–34,806)	218 (4–1647)	−3.591	< 0.001
Kidney disease	12,530 (3793–16,519)	153 (96–411)	−3.000	0.003
p	< 0.001	< 0.001		
Number of complications				
1	3486 (1813–6479)	68 (22–195)	−42.365	< 0.001
2	1849 (870–3900)	91 (38–243)	− 28.406	< 0.001
3	1805 (962–4072)	150 (70–390)	−21.015	< 0.001
4	2610 (1404–6300)	469 (219–2124)	−10.150	< 0.001
5	3517 (1722–12,818)	1937 (370–7832)	−3.764	< 0.001
6	3984 (1893–17,492)	1509 (710.60–11,237)	− 3.211	0.001
7	6310 (2934–21,999)	8697 (1260–39,388)	− 0.26	0.979
8	5732 (3052–25,133)	22,091 (4553–42,832)	−1.607	0.108
9	6691 (2758–40,848)	18,297 (7526–59,551)	−1.248	0.212
≥ 10	13,962 (4995–41,706)	44,324 (14191–89,266)	−4.156	< 0.001
p	< 0.001	< 0.001		
Payment type				
Social Security Card	4083 (1892–11,676)	71 (32–186)	−33.888	< 0.001
Medicare Card	2155 (1080–5345)	64 (27–157)	−26.332	< 0.001
Hospital Link Card	3278 (1503–8006)	90 (27–274)	− 58.033	< 0.001
Hospital Self-Charge Card	3506 (1415–14,791)	287 (117–892)	−6.936	< 0.001
Others	1363 (837–3399)	2158 (570–12,708)	− 0.234	0.815
p	< 0.001	< 0.001		
Total	2953 (1425–7028)	79 (30–228)	−81.944	< 0.001

### Estimation of economic burden

We employed a generalized linear model to estimate the direct medical cost for inpatients, outpatients, and overall. The inpatient model included variables which were shown significant from the stratified analysis, and was estimated based on the features of age, disease type, number of complications, and payment type. Analogously, the fitted model for outpatients was built on the features of age, gender, disease type, number of complications, and payment type.

### Mean cost

The mean annual cost of inpatients was estimated as ¥9846.77(95%CI, ¥8371.44–¥10,689.97) (US$1562.97). The estimated mean annual cost of outpatients was ¥1047.26(95%CI, ¥940.40–¥1154.11) (US$166.19).

### Factors (age, complication, disease type, payment type)

Additionally, the annual direct cost for the following features (age, gender, disease type, number of complication, and payment type), separated by inpatients (Table 3) and outpatients (Table 4) was estimated as well. Below we highlight some key aspects and differences between the inpatient and outpatient predictions.

**Table 3:** Estimated direct medical cost (¥) of inpatients with different characteristics

Parameter	B	P*	Estimated Mean (95% CI)	P**
Age group (years)				
≤ 14	Ref.	N.A.	12,923 (10315–16,191)	< 0.001
15~64	.0210	< 0.001	15,951 (12879–19,756)	0.766
≥ 65	−0.385	< 0.001	8794 (6781–11,405)	0.016
Disease type				
Endocrine and metabolic disease	Ref.	N.A.	3866 (3286–4547)	< 0.001
Skin disease	1.258	< 0.001	13,602 (7139–25,916)	< 0.001
Blood disease	1.530	< 0.001	17,856 (15581–20,463)	0.019
Digestive disease	0.930	< 0.001	9799 (8030–11,960)	< 0.001
Bone disease	1.165	< 0.001	12,395 (8022–19,152)	< 0.001
Cardiovascular disease	0.827	< 0.001	8839 (7344–10,638)	< 0.001
Immunological disease	2.148	< 0.001	33,107 (14863–73,745)	0.019
Kidney disease	1.332	0.001	14,639 (6601–32,465)	0.118
Number of complications				
1	Ref.	N.A.	7527 (5995–9452)	< 0.001
2	−0.267	< 0.001	5762 (4585–7240)	0.676
3	−0.369	< 0.001	5202 (4146–6528)	0.577
4	0.084	0.331	8190 (6430–10,431)	0.916
5	0.313	0.001	10,290 (7959–13,304)	0.270
6	0.538	< 0.001	12,891 (9809–16,942)	0.729
7	0.886	< 0.001	18,259 (13434–24,817)	0.950
8	0.955	< 0.001	19,556 (14209–26,913)	0.960
9	1.175	< 0.001	24,386 (16946–35,085)	0.556
> 10	1.509	< 0.001	34,029 (25593–45,245)	0.620
Payment type				
Social Security Card	Ref.	N.A.	10,567 (8663–12,890)	0.002
Medicare Card	−.280	0.001	7986 (6423–9930)	0.746
Hospital Link Card	0.005	0.934	10,623 (8821–12,794)	0.466
Hospital Self-Charge Card	1.396	< 0.001	42,691 (27803–65,550)	0.611
Others	−0.406	0.060	7041 (4491–11,038)	< 0.001

*P value for coefficient B

**P value for estimated mean compared with observed mean

**Table 4:** Estimated direct medical cost (¥) of outpatients with different characteristics

Parameter	B	P*	Estimated Mean (95% CI)	P**
Age group (years)				
≤ 14	Ref.	N.A.	5235 (2398–11,427)	< 0.001
15~64	−0.753	< 0.001	2466 (1136–5354)	0.056
≥ 65	−1.471	< 0.001	1202 (545–2651)	< 0.001
Gender				
Female	Ref.	N.A.	2153 (983–4716)	< 0.001
Male	0.294	< 0.001	2889 (1331–6274)	0.910
Disease type				
Endocrine and metabolic disease	Ref.	N.A.	2902 (1524–5526)	< 0.001
Skin disease	0.020	0.959	2961 (1100–7969)	< 0.001
Blood disease	1.089	< 0.001	8623 (4537–16,390)	< 0.001
Digestive disease	0.834	< 0.001	6681 (3483–12,813)	< 0.001
Bone disease	−0.141	0.773	2521 (799–7954)	< 0.001
Cardiovascular disease	0.701	< 0.001	5847 (2915–11,728)	< 0.001
Immunological disease	−3.927	0.010	57 (3–1233)	0.110
Kidney disease	0.213	0.756	3591 (809–15,935)	< 0.001
Number of complications				
1	Ref.	N.A.	160 (76–338)	< 0.001
2	0.445	< 0.001	250 (118–529)	< 0.001
3	1.668	< 0.001	847 (393–1830)	0.234
4	1.784	< 0.001	952 (419–2163)	0.003
5	2.621	< 0.001	2199 (903–5358)	0.001
6	3.633	< 0.001	6051 (2180–16,797)	0.336
7	3.602	< 0.001	5864 (1954–17,597)	0.003
8	3.892	< 0.001	7835 (2133–28,780)	0.024
9	4.718	< 0.001	17,899 (4859–65,922)	0.128
> 10	5.108	< 0.001	26,452 (9963–70,228)	< 0.001
Payment type				
Social Security Card	Ref.	N.A.	2696 (1800–4897)	< 0.001
Medicare Card	−1.044	< 0.001	1045 (634–1724)	0.006
Hospital Link Card	−0.579	< 0.001	1664 (1015–2726)	< 0.001
Hospital Self-Charge Card	−0.110	0.395	2660 (1550–4565)	< 0.001
Others	0.862	0.574	7032 (335–147,832)	0.690

*P value for coefficient B

**P value for estimated mean compared with observed mean

#### Age

Regarding age, for inpatients, it was estimated that adult (age 15–64) would incur highest direct costs: ¥15,950.75 (95%CI; ¥12,878.64 - ¥19,755.69) per year of all 3 age groups. For the outpatients, the children group (age ≤ 14) had the highest predicted cost (¥5234.71; 95%CI; ¥2398.12–¥11,426.50) than the other groups.

#### Disease type

In the inpatients model, the top 3 disease categories by predicted annual cost were immunological, blood, and kidney disease. The immunological category stood out in terms of predicted annual cost which was estimated to be ¥33,107.45(¥14,863.49–¥73,744.67), far surpassing other disease types (almost twice the level of the second one in the ranking). The lowest predicted annual cost was in endocrine-metabolic disease ¥3865.73 (¥3286.41–¥4547.17). For outpatients group the top 3 disease categories by predicted annual cost were blood, digestive, and cardiovascular disease. The diseases in the blood were estimated to be ¥8623.34(¥4537.17–¥16,389.50). The lowest predicted annual cost was in immunological disease ¥57.19(¥2.65–¥1232.87). Notable in our model is that diseases from the immunological category showed high annual cost in inpatients but low annual cost in outpatients, whereas for blood disease predicted cost were high for both inpatient and outpatient groups.

#### Complications

Our model showed a trend that a higher cost associated with an increasing number of complications. The number of complication levels (1 to 10+) were significantly different in cost for both actual and estimated data in both inpatient and outpatient groups.

#### Payment type

For inpatients with hospital self-charge card (*zifeika*, 自费卡), (which is not subject to insurance reimbursement) annual expenditure was estimated as the highest in all 4 payment types. Annual estimated cost - ¥42,691.14(¥27,803.37–¥65,550.80) for such individuals was over 4 times higher than the second one in the list ranking. The Medicare card (*yibaoka*, 医保卡) type had the lowest predicted level ¥7986.49(¥6423.07–¥9930.45). For outpatients, hospital self-charge card (*zifeika*, 自费卡) and social security card (*shebaoka*, 社保卡) were at nearly the same levels, ¥2659.64(¥1549.57–¥4564.95) and ¥2696.02(¥1799.94–¥4897.43), respectively. Notable is that inpatients had a much higher level of predicted annual cost as compared to outpatients.

#### Direct cost

For a total population of 5185 inpatients and 11,748 outpatients, computed from model estimates, the annual medical expenditure for patients with the 23 rare diseases in Shanghai were estimated to be ¥15,839,678 (US$2,514,235).

## Discussion

Globally, rare disease management presents a significant challenge [19] to health policy makers, health care providers, patients, and society in general due to difficulties of treatment, gaps in knowledge, cost, and drug access; to name a few. To successfully address these unique issues, integrated efforts of all participants inside the health care system and continuous research efforts on the many unanswered questions ranging from basic science to policy, are required [20]. This work adds a China-focused contribution to rare disease research and helps bring further awareness of rare disease impact on Chinese society. We measured the direct cost attributable to rare diseases with effective treatments for outpatient and inpatients in hospitals and medical care centers in Shanghai. The inpatient and outpatient stratification of each disease population underlies a key aspect of our study which sets it apart from existing work. Specifically, and with the goal of comprehensive overview, we analyzed economic burden based not only on inpatient cost, but also on outpatient cost. It should be noted that there are certain limitations of the analysis in the work which stem from potential sampling issues due to the well-known difficulty of rare disease diagnosis, thus certain disease associated costs may be underestimated.

The distribution of costs over patients is characterized by positive (right) skewness, with the median cost lower than the mean cost in our study, indicating a small proportion of patients incurring much higher costs. For example, patients with more than ten complications were incurring costs at least five times more than those of patients with one complication. Similar trend was observed in recent systematic review of cost of illness study over 10 rare diseases in Europe [15]. Additionally, in the summary of our estimated economic burden, we reported the mean cost rather than the median, which can allow better interpretation of the variation in severity among patients. Due to this right skewness feature of rare diseases cost distribution, the calculated cost may appear affordable for certain patients. However, taking some outliers as example, a dramatically different picture is revealed - patients diagnosed with severe congenital neutropenia spent over ¥1.5 million ($238,000) in total in 2016. For these patients paying for treatment can be financially catastrophic and may be unaffordable if self-funded. Receiving treatment in such cases is therefore dependent on whether costs incurred by the patient will be covered by a public or private health insurance program.

Furthermore, we draw a comparison of rare disease treatment cost vs. annual disposable income in Shanghai. The annual average disposable income of Shanghai in 2015 was ¥49,867($7915), further stratified into ¥52,962($8406) for central urban area residents and ¥23,205($3683) for residents in countryside and rural areas as reported by the Shanghai government [21]; for China nationwide, the overall average was ¥21,966 ($3486); ¥31,195 ($4951) for central urban area residents, and ¥11,422 ($1813) countryside/rural areas residents [22]. Compared to the annual disposable income, cost of rare disease treatment for inpatients constituted a large proportion of annual disposable income for central urban area resident and almost half the annual disposable income for rural area residents of Shanghai. Additionally, the direct inpatient medical cost (¥9588, US$1521) was found far surpassing per capita consumption expenditure on medical care, which is ¥2268($360) in 2015 [21] for Shanghai, and ¥1165($184) nationwide [22]. Therefore, the direct cost of rare diseases is a heavy burden for a family, especially one from the countryside and rural areas.

Management of rare disease is a complex and multifaceted problem requiring increased awareness, patient tracking, and cost control. In recent year, steps have been implemented to address these aspects. The National Rare Diseases Registry System of China was launched in 2016 [23] and a proportion of drugs used in rare diseases have been covered in the medical reimbursement system in some provinces. Since 2011, drugs for Pompeii disease, Gaucher’s disease, mucopolysaccharidosis, and Fabry disease have been covered by the Children’s Hospitalization Fund in Shanghai [24]. Additionally, further efforts will be needed to address holistically the issue of drug treatment costs which will continue to impose significant economic burden [25] on Chinese patients, families, and society in general.

## Conclusions

This study is focused on the economic burden of rare diseases with effective treatments in Shanghai. From statistical analysis, we found the cost of illness correlated with patient age, disease type, disease severity, and payment type. In addition, we described the actual direct medical expenditure and estimated the socioeconomic cost being of over ¥15 million. Attention to rare diseases from therapeutic and governance perspective has continued to increase in China, further driving public awareness and incentivizing development of rare-disease strategies and treatments specific to the Chinese context. Further challenges however still remain; therefore, close collaboration between pharmaceutical companies, patients, medical care providers, insurance companies, and regulatory authorities would be needed to achieve an environment where the best treatment is provided for rare disease patients in China, while at the same time accounting for the non-trivial cost imposed by the treatment and management of rare diseases.

## Additional file

Additional file 1: **Table S1.** The list of major rare diseases in Shanghai. **Table S2** Composition of direct medical cost. **Table S3.** Sample size and mean cost of 34 rare diseases in Shanghai, China. **Table S4.** Payment types and coverage options for medical treatment in Shanghai, China. (DOCX 36 kb)

## Data Availability

Not applicable.
