# Supplementary material for: Analysis of economic burden and its associated factors of twenty-three rare diseases in Shanghai
Source: Orphanet J Rare Dis. 2019 Oct 22;14:233. doi: 10.1186/s13023-019-1168-4 (PMC6806581; doi:10.1186/s13023-019-1168-4)
Supplement: Supplementary file 1 — Table S1. The list of major rare diseases in Shanghai. Table S2 Composition of direct medical cost. Table S3. Sample size and mean cost of 34 rare diseases in Shanghai, China. Table S4. Payment types and coverage options for medical treatment in Shanghai, China. (DOCX 36 kb) [file 13023_2019_1168_MOESM1_ESM.docx]

Additional file 1

**Table S1.** The list of major rare diseases in Shanghai

| **NO** | **CATEGORY** | **NAME OF DISEASE** |
| --- | --- | --- |
| 1 | Kidney disease | Atypical hemolytic uremic syndrome |
| 2 | Endocrine and metabolic disease | Biotinidase deficiency |
| 3 | Endocrine and metabolic disease | citrullinemia |
| 4 | Endocrine and metabolic disease | Congenital adrenal hyperplasia |
| 5 | Endocrine and metabolic disease | Congenital hperinsulinemic hypoglycemia |
| 6 | Blood disease | Diamond-Blackfan anemia |
| 7 | Endocrine and metabolic disease | Fabry disease |
| 8 | Blood disease | Fanconi anemia |
| 9 | Endocrine and metabolic disease | Galactosemia |
| 10 | Endocrine and metabolic disease | Gaucher's disease |
| 11 | Endocrine and metabolic disease | Glutaric acidemia I |
| 12 | Endocrine and metabolic disease | Glycogen storage diseases |
| 13 | Blood disease | Hemophilia |
| 14 | Digestive disease | Hepatolenticular degeneration |
| 15 | Skin disease | Hereditary epidermolysis bullosa |
| 16 | Bone disease | Hypophosphatasia |
| 17 | Bone disease | Hypophosphatemic rickets |
| 18 | Cardiovascular disease | Idiopathic pulmonary arterial hypertension |
| 19 | Endocrine and metabolic disease | Laron syndrome |
| 20 | Endocrine and metabolic disease | Maple syrup urine disease |
| 21 | Endocrine and metabolic disease | Methylmalonic acidemia |
| 22 | Endocrine and metabolic disease | Mucopolysaccharidoses |
| 23 | Endocrine and metabolic disease | Niemann-Pick disease |
| 24 | Endocrine and metabolic disease | Noonan syndrome |
| 25 | Bone disease | Osteogenesis imperfecta |
| 26 | Endocrine and metabolic disease | Phenylketouria |
| 27 | Endocrine and metabolic disease | Prader-Willi syndrome |
| 28 | Endocrine and metabolic disease | Primary carnitine deficiency |
| 29 | Endocrine and metabolic disease | Propionic acidemia |
| 30 | Blood disease | Severe congenital neutropenia |
| 31 | Endocrine and metabolic disease | Silver-Russell syndrome |
| 32 | Endocrine and metabolic disease | Tyrosinemia |
| 33 | Immunological disease | Wiskott-Aldrich syndrome |
| 34 | Immunological disease | X-linked lymphoproliferative disease |

**Table S2.** Composition of direct medical cost

| **Code** | **Chinese name** | **English name** | **Code** | **Chinese name** | **English name** |
| --- | --- | --- | --- | --- | --- |
| 00 | 挂号费 | Registration cost | 08 | 摄片费 | Imaging cost |
| 01 | 住院费 | Hospitalization cost | 09 | 透视费 | - Fluoroscopy   cost |
| 02 | 诊疗费 | Diagnostic cost | 10 | 输血费 | Blood transfusion cost |
| 03 | 治疗费 | Treatment cost | 11 | 输氧费 | Supplementary Oxygen cost |
| 04 | 护理费 | Nursing services cost | 12 | 西药费 | Conventional medicine cost |
| 05 | 手术材料费 | Surgical materials cost | 13 | 中成药费 | Traditional Chinese medicine (pill form) cost |
| 06 | 检查费 | Inspection cost | 14 | 中草药费 | Traditional Chinese medicine (herbal drink form) cost |
| 07 | 化验费 | Laboratory cost | 15 | 其他费用 | Other expenses |


**Table S3.** Sample size and mean cost of 34 rare diseases in Shanghai, China

| **NO** | **NAME OF DISEASE** | **ICD10 CODE** | **SAMPLE SIZE**  **(Number of patients with cost information entered into the HIE system)** | **MEAN COST(**¥**)** |
| --- | --- | --- | --- | --- |
| 1 | Atypical hemolytic uremic syndrome | D59.301 | 16 | 13486.63 |
| 2 | Biotinidase deficiency | D81.801 | NA | NA |
| 3 | Citrullinemia | E72.202 | NA | NA |
| 4 | Congenital adrenal hyperplasia | E25.004 | 856 | 13343.55 |
| 5 | Congenital hperinsulinemic hypoglycemia | E16.103 | 3507 | 1454.37 |
| 6 | Diamond-Blackfan anemia | D61.001 | 36 | 10986.67 |
| 7 | Fabry disease | E75.205 | 55 | 1891.98 |
| 8 | Fanconi anemia | E72.002 | 60 | 16030.11 |
| 9 | Galactosemia | E74.201 | NA | NA |
| 10 | Gaucher's disease | E75.201 | 158 | 1619.09 |
| 11 | Glutaric acidemia I | E72.302 | NA | NA |
| 12 | Glycogen storage diseases | E74.000 | 363 | 1406.91 |
| 13 | Hemophilia | D66.x01/D66.x02/  D67.x01/D68.101 | 1823 | 35692.67 |
| 14 | Hepatolenticular degeneration | E83.001 | 1532 | 6828.40 |
| 15 | Hereditary epidermolysis bullosa | Q81.900 | 174 | 5848.99 |
| 16 | Hypophosphatasia | E83.306 | NA | NA |
| 17 | Hypophosphatemic rickets | E83.308 | NA | NA |
| 18 | Idiopathic pulmonary arterial hypertension | I27.000 | 1078 | 16438.84 |
| 19 | Laron syndrome | E34.304 | NA | NA |
| 20 | Maple syrup urine disease | E71.001 | 71 | 1469.71 |
| 21 | Methylmalonic acidemia | E71.102 | 92 | 23730.12 |
| 22 | Mucopolysaccharidoses | E76.000 | NA | NA |
| 23 | Niemann-Pick disease | E75.203 | 34 | 20189.86 |
| 24 | Noonan syndrome | Q87.105 | NA | NA |
| 25 | Osteogenesis imperfecta | Q78.000 | 152 | 6604.24 |
| 26 | Phenylketouria | E70.000 | 54 | 4373.73 |
| 27 | Prader-Willi syndrome | Q87.106 | 141 | 10732.92 |
| 28 | Primary carnitine deficiency | E71.302 | 15 | 21542.45 |
| 29 | Propionic acidemia | E71.101 | 22 | 12294.99 |
| 30 | Severe congenital neutropenia | D70.x00 | 5345 | 17640.65 |
| 31 | Silver-Russell syndrome | Q87.100 | NA | NA |
| 32 | Tyrosinemia | P74.501 | NA | NA |
| 33 | Wiskott-Aldrich syndrome | D82.000 | 20 | 51056.60 |
| 34 | X-linked lymphoproliferative disease | D82.301 | 10 | 16012.52 |

**Table S4.** Payment types and coverage options for medical treatment in Shanghai, China

| **Payment** | ***Chinese Name*** | **Coverage Type** | **Demographics** | **Additional info** |
| --- | --- | --- | --- | --- |
| social security card | *shebaoka*  社保卡 | Medical Insurance Coverage | People with Shanghai permanent residence (hukou) | This card is issued by the Shanghai municipal government to holders of Shanghai permanent residence (hukou). Medical cost exceeding the co-pay amount are covered by the insurance plan |
| medicare card | *yibaoka*  医保卡 | Medical Insurance Coverage | People with Shanghai residence permit | This card is issued by the Shanghai municipal government to holders of Shanghai residence permit holder. Medical cost exceeding the co-pay amount are covered by the insurance plan |
| hospital link card | *yilianka*  医联卡 | Self-funded | Can be obtained by anyone | This card serves as a patient identifier and payment method after delivery of medical services. Upon registration in the hospital that is a member of the hospital link system a patients is issued this card which is tied to a medical record and a monetary amount pre-paid by the patient. The patient use the card to access individual health information, test results , etc. |
| hospital self-charge card | *zifeika*  自费卡 | self-funded | Can be obtained by anyone | This is the most basic form. Upon registration in the hospital the patient is issued such card which is tied to a medical record and stored monetary value. |
